# N‐terminal pro‐brain natriuretic peptide is a prognostic marker for response to intensive chemotherapy, early death, and overall survival in acute myeloid leukemia

**DOI:** 10.1002/ajh.26805

**Published:** 2023-01-01

**Authors:** Irene Graf, Georg Greiner, Rodrig Marculescu, Karoline V. Gleixner, Susanne Herndlhofer, Gabriele Stefanzl, Paul Knoebl, Ulrich Jäger, Alexander Hauswirth, Ilse Schwarzinger, Renate Thalhammer, Michael Kundi, Gregor Hoermann, Gerlinde Mitterbauer‐Hohendanner, Peter Valent, Wolfgang R. Sperr

**Affiliations:** ^1^ Division of Hematology and Hemostaseology, Department of Internal Medicine I Medical University of Vienna Vienna Austria; ^2^ Department of Laboratory Medicine Medical University of Vienna Vienna Austria; ^3^ Ludwig Boltzmann Institute for Hematology and Oncology Medical University of Vienna Vienna Austria; ^4^ Ihr Labor, Medical Diagnostic Laboratories Vienna Austria; ^5^ Comprehensive Cancer Center Vienna Medical University of Vienna Vienna Austria; ^6^ Institute of Environmental Health Medical University of Vienna Vienna Austria; ^7^ MLL Munich Leukemia Laboratory Munich Germany

## Abstract

Patient‐related factors are of prognostic importance in acute myeloid leukemia (AML). Likewise, cardiac disorders may limit the tolerance of intensive therapy. Little is known about the prognostic value of N‐terminal pro‐brain natriuretic peptide (NT‐proBNP). We analyzed NT‐proBNP levels at diagnosis in 312 AML patients (median age: 61 years; range 17–89 years) treated with 3 + 7‐based induction‐chemotherapy and consolidation with up to four cycles of intermediate or high‐dose ARA‐C. NT‐proBNP levels were elevated in 199 patients (63.8%), normal (0–125 pg/ml) in 113 (36.2%), and highly elevated (>2000 pg/ml) in 20 patients (6.4%). Median NT‐proBNP levels differed significantly among patients with complete remission (153.3 pg/ml), no remission (225.9 pg/ml), or early death (735.5 pg/ml) (*p* = .002). In multivariate analysis, NT‐proBNP, age, and the 2009 European LeukemiaNet (ELN‐2009) classification were independent predictors of outcome after induction chemotherapy. Overall survival (OS) differed significantly between patients with normal, moderately elevated, and highly elevated NT‐proBNP (*p* < .001). These differences were observed in all patients and in patients <60 years but not in those ≥60 years. In multivariate analysis, NT‐proBNP, age, and ELN‐2009 remained independent prognostic variables for OS (*p* < .01). Together, NT‐proBNP is an independent prognostic factor indicating the risk of induction failure, early death, and reduced OS in patients with AML.

## Graphical abstract

Overall survival differed significantly among patients with normal N‐terminal pro brain natriuretic peptide (NT‐proBNP; <125 μg/mL), those with moderately elevated (125–200 pg/mL), and those with highly elevated levels of NT‐proBNP (>2000 pg/mL) as assessed by log rank test.

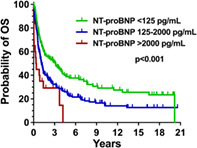

## 1 INTRODUCTION

Acute myeloid leukemia (AML) is a hematologic malignancy characterized by abnormal proliferation of neoplastic myeloid precursor cells and a maturation defect.^1^, ^2^ Despite the availability of curative chemotherapies and hematopoietic stem cell transplantation (HSCT), the prognosis in patients with AML remains poor. Death within the first 60 days after initiation of induction therapy is seen in a substantial number of patients and long‐term survival can only be achieved using intensive induction chemotherapy with conventional consolidation or HSCT.^3^, ^4^, ^5^, ^6^ However, even after chemotherapy and HSCT, patients with AML may relapse. Several different disease‐related parameters are of prognostic value in AML, including the karyotype and molecular aberration profiles at diagnosis.^2^, ^6^ These variables have been included in various risk scores and stratification models.

Apart from disease‐related variables, several patient‐related factors, including age, poor organ function and especially cardiac comorbidities are prognostic factors in patients with AML.^7^, ^8^, ^9^ These variables are of particular importance when exploring the ability of patients to tolerate high‐dose induction chemotherapy and/or HSCT. Based on relevant comorbidities, several scoring systems for clinical risk stratification, including the Eastern Cooperative Oncology Group (ECOG) and Charlson comorbidity index (CCI) have been established.^7^, ^8^ However, such comorbidities and their actual impact are often not visible at first glance or are not known. Therefore, cardiac ultrasound or multi‐gated acquisition scan are often recommended to assess organ function especially in elderly patients before chemotherapy.^9^, ^10^, ^11^ In this regard, it should also be noted, that in some patients with AML and acute problems such as severe infections or hyperleukocytosis, a detailed medical checkup before initiating therapy is not always possible. So far, a robust laboratory‐based biomarker assessing cardiac function and predicting tolerability of chemotherapy and general outcomes is lacking in AML.

NT‐proBNP is an important biomarker and predictor of cardiac morbidity and mortality and has further been considered to play a prognostic role in various non‐cardiologic diseases such as diabetes mellitus, severe septicemia, amyloidosis, and in certain hematologic diseases, like sickle cell disease or non‐Hodgkin lymphoma (NHL).^12^, ^13^, ^14^, ^15^, ^16^, ^17^, ^18^, ^19^, ^20^, ^21^, ^22^, ^23^

The aim of the current study was to determine the prognostic value of serum NT‐proBNP concentrations in patients with AML at diagnosis. We evaluated NT‐proBNP levels in 312 patients with AML. We found that the NT‐proBNP level at diagnosis is of prognostic significance, predicting responses to chemotherapy, early death, and survival in these patients.

## 2 PATIENTS AND METHODS

### 2.1 Patients

We collected disease‐ and patient‐related parameters in 312 patients with AML (median age: 61 years; range 17–89 years; f:m‐ratio: 1:1.15). The characteristics of these patients are shown in Table 1. The data were obtained retrospectively by chart review at diagnosis and during follow up. Patients were diagnosed with de novo AML (*n* = 260) or secondary AML (*n* = 52) at the Medical University of Vienna between February 1998 and November 2020. Patients were diagnosed and classified according to the 2016 version of the World Health Organization classification of myeloid neoplasms and acute leukemia.^24^ Patients with t(15;17) positive AML (promyelocytic leukemia) were excluded from the analysis. All patients provided written informed consent. The study was approved by the ethics committee of the Medical University of Vienna (EK‐number: 1184/2014).

**TABLE 1:** AML patients' characteristics

Variable	All patients	Pts with NT‐proBNP ≤125	Pts with NT‐proBNP >125	*p*‐value*
Number of pts	312	113	199	
Sex (male/female), *n* (%)	167 (53.5%)/ 145 (46.5%)	61 (54%)/52 (46%)	106 (53.3%)/ 93 (46.7%)	.903
f:m‐ratio	1:1.15	1:1.17	1:1,14	
Age				
Median (IQR)	61 (21)	57 (24)	63 (20)	.003
Pts aged <60 years, *n* (%)	141 (45.2%)	63 (20.2%)	78 (25.0%)	.006
Pts aged ≥60 years, *n* (%)	171 (54.8)	50 (16.0%)	121 (38.8%)	
ECOG (*n* = 310)				.027
0 points	214 (69.0%)	89 (28.7%)	125 (40.3%)	
1 point	89 (28.7%)	23 (7.4%)	66 (21.4%)	
2 points	5 (1.6%)	0 (0%)	5 (1.6%)	
3 points	2 (0.7%)	0 (0%)	2 (0.6%)	
CCI (*n* = 289)				<.001
Low risk	59 (20.4%)	59 (20.4%)	0 (0.0%)	
Moderated risk	79 (27.3%)	50 (17.3%)	29 (10.0%)	
Intermediate risk	95 (32.9%)	16 (5.5%)	79 (23.3%)	
High risk	56 (19.4%)	5 (1.7%)	51 (19.4%)	
ELN (*n* = 290)				.483
Favorable	69 (23.8%)	23 (7,9%)	46 (15.9%)	
Intermediate 1	106 (36.5%)	41 (14.1%)	65 (22.4%)	
Intermediate 2	58 (20%)	21 (7.2%)	37 (12.8%)	
Adverse	57 (19.7%)	17 (5.9%)	40 (13.8%)	
Year of AML diagnosis, median (IQR) (*n* = 312)	2006 (13)	2005 (12.5)	2006 (14)	.935
Treatment type (*n* = 312)				.863
Daunorubicin 45 mg/m^2^, *n* (%)	183 (58.6%)	67 (21.5%)	116 (37.2%)	
Daunorubicin 60 mg/m^2^, *n* (%)	129 (41.4%)	46 (14.7%)	83 (26.6%)	
Baseline cardiac function				.007
Normal LVF	172 (91.5%)	68 (100%)	104 (86.7%)	
Reduced LVF	16 (8.5%)	0 (0%)	16 (13.3%)	
WBC (×10^3^), median (IQR) (*n* = 312)	10.84 (43.6)	4.53 (16.98)	16.38 (51.45)	<.001
FBG (mg/dl), median (IQR) (*n* = 302)	398 (194)	381 (187)	406 (203)	.448
CRP (mg/dl), median (IQR) (*n* = 312)	2.3 (6.9)	1.1 (4.1)	3.4 (7.6)	<.001
Albumin (mg/dl), median (IQR) (*n* = 270)	37.8 (8.5)	39.9 (9.8)	36.8 (8.7)	.004
Creatinine (mg/dl), median (IQR) (*n* = 274)	0.97 (0.29)	0.87 (0.26)	1.02 (0.34)	<.001
LDH (U/L), median (IQR) (*n* = 310)	410 (506)	296 (322)	484 (533)	<.001

Abbreviations: CCI, Charlson comorbidity index; CRP, C‐reactive protein; ECOG, Eastern Cooperative Oncology Group performance status; ELN, European leukemia network risk classification 2009; FBG, fibrinogen; IQR, interquartile range; LDH, lactate dehydrogenase; LVF, left ventricular function; n, number of patients; pts, patients; WBC, white blood cell count.

* The significance of differences were assessed by Mann‐Whitney and Kruskal Wallis tests for the comparison of two groups and more than two groups, respectively. For comparing categorical variables, the Chi‐square test was applied.

### 2.2 Treatment of AML

For induction, a daunorubicin and ARA‐C (3 + 7)‐based induction chemotherapy protocol was employed.^25^ Until October 2016, all patients, regardless of age, received DAV 3 + 5 + 7 (daunorubicin, 45 mg/m^2^ iv, days 1–3; etoposide 100 mg/m^2^ iv, days 1–5; ARA‐C 2 × 100 mg/m^2^ iv, days 1–7). After October 2016, a DA 3 + 7 protocol (daunorubicin 60 mg/m^2^ iv, days 1–3; ARA‐C 100 mg/m^2^ iv, continuous infusion, days 1–7) was administered in all patients. In patients with *FLT3*‐mutated AML, midostaurin (2 × 50 mg po, starting at the 1st day after the end of chemotherapy until the day before the 1st day of the next chemotherapy‐cycle; and for 336 days from the day after the last chemotherapy) or gemtuzumab‐ozogamicin = GO (3 mg/m^2^ iv, days 1, 4, 7 during induction and on day 1 of consolidations 1 and 2) was added. In case of blast cell persistence after the first induction cycle, a second induction cycle was given. Patients <60 years received MiDAC (ARA‐C, 2 × 1 g/m^2^ iv, days 1–4; mitoxantrone 12 mg/m^2^ days 3–5).^26^ In patients aged ≥60 years, DAV 2 + 5 + 5 (daunorubicin, 45 mg/m^2^ iv, days 1–2; etoposide 100 mg/m^2^ iv, days 1–5; ARA‐C 2 × 100 mg/m^2^ iv, days 1–5) was given until July 2008. After July 2008, a modified MiDAC protocol (ARA‐C, 2 × 1 g/m^2^ iv, days 1, 3, 5; mitoxantrone, 12 mg/m^2^ iv, days 3, 5) was applied in these patients.^27^ Response to treatment was assessed after induction: patients in complete remission (CR) after the first or second induction cycle were included as CR patients. After CR, up to four cycles of consolidation therapy were applied: patients below 60 years received up to four cycles of high‐dose ARA‐C (2 × 3 g/m^2^ iv, days 1, 3, and 5 or days 1, 2, and 3; HiDAC), patients ≥60 were treated with intermediate dose ARA‐C (2 × 1 g/m^2^ iv, days 1, 3, and 5 or days 1, 2, and 3; IDAC).^4^, ^28^ From August 2008, induction chemotherapy was intensified markedly in patients ≥60 years by including a modified MiDAC protocol as second induction and FLAG (fludarabine, 30 mg/m^2^ iv, days 1–5; ARA‐C 2 g/m^2^ iv, days 1–5; granulocyte/macrophage colony‐stimulating factor, G‐CSF, from day 6 until neutrophil recovery) as first consolidation (Table S1).^29^

### 2.3 Evaluation of risk factors and comorbidities

We extracted clinical data on comorbidities from the patients' charts for calculation of the CCI.^29^ Based on CCI assessment, we stratified our patients into those without comorbidities (0 points), those with moderate risk (1–2 points), intermediate risk (3–4 points), or high‐risk (≥5 points) (Table S2).^30^ The karyotype and information on *NPM1* and *FLT3* mutations were available in almost all patients. Information on other molecular lesion was only available in subset of patients. Therefore, we used the ELN‐2009 risk‐based classification of AML.^31^ Moreover, the Eastern Cooperative Oncology Group (ECOG) performance status was applied.^32^ Patient were classified as: ECOG 0, “fully active, able to carry on all pre disease performance without restriction”; ECOG 1, “restricted in physically strenuous activity, but ambulatory and able to carry out work of a light and sedentary nature”; ECOG 2, “ambulatory and capable of all self‐care but unable to carry out any work activities; up and about more than 50% of waking hours”; ECOG 3, “capable of only limited self‐care, confined to bed or chair more than 50% of waking hours”; ECOG 4, “completely disabled, cannot carry on any self‐care, totally confined to bed or chair”; and ECOG 5, “dead”.^32^

### 2.4 Measurement of NT‐proBNP levels in the sera of patients with AML

We applied an immunoassay for measuring serum NT‐proBNP levels (Roche, Basel, Switzerland) on a Cobas e601 immunochemistry module (Roche) according to the instructions of the manufacturer. Starting in 2016, NT‐proBNP levels were measured routinely at diagnosis in our AML patients. Moreover, we used serum samples stored in a local biobank for measuring serum NT‐proBNP levels that had not been determined at diagnosis. In brief, the serum tubes were centrifuged for 10 min at 1882×*g*. Thereafter, serum samples were stored at −20°C. Normal NT‐proBNP serum levels range between 0 and 125 pg/ml in the assay applied.^14^ As shown in Figure S1, serum NT‐proBNP levels remained in the same range when comparing samples measured at the time of collection or after the samples had been stored for 4–5 years, >5–10 years, >10–15 years, or >15–22 years at −20°C, and NT‐proBNP levels were determined after freeze‐thawing. Notably, no substantial decrease in the median NT‐proBNP level was found over time in these patients (*p* = .169 by ANOVA).

### 2.5 Statistical analysis

Differences in NT‐proBNP levels were evaluated for statistical significance by Mann‐Whitney and Kruskal Wallis tests for comparing two groups and more than two groups, respectively, in univariate analyses. For comparing categorical variables, the Chi‐square test was applied. For multivariate analysis, evaluating the impact of prognostic variables on treatment responses, a general linear model was used. We applied cubic splines of NT‐proBNP to analyze the relationship to overall survival (OS). The first knot was significant indicating a log‐linear relationship. For easier interpretation, we chose a subdivision into three categories, that is, patients with normal NT‐proBNP levels (0–125 pg/ml), elevated NT‐proBNP levels (125–2000 pg/ml), and highly elevated NT‐proBNP levels (>2000 pg/ml). The second cut off was chosen based on data showing that in patients with clinical signs of heart failure, NT‐proBNP levels above 1800 pg/ml are considered as highly indicative of cardiac insufficiency.^12^, ^13^, ^14^, ^15^

Early death (ED) was defined as death after induction therapy within 60 days. The product limit method of Kaplan and Meier was used to analyze the probability of OS and relapse‐free survival (RFS). OS was defined as time from start of therapy to death. Patients still at risk were censored. RFS was defined as time from achieving a complete remission (CR) to relapse or death. Patients still at risk were censored. The probability of OS and RFS were analyzed with and without censoring patients receiving HSCT. Differences in survival among cohorts were assessed by log rank test. Multivariate analyses were performed by Cox regression. Differences were considered significant with a *p*‐value < .05. Statistical calculations were carried out in Microsoft Excel for MAC Version 16.16 and IBM SPSS Statistics for MAC Version 25.0.

## 3 RESULTS

### 3.1 NT‐proBNP levels in AML patients

The median serum NT‐proBNP concentration in our group of 312 AML patients was 191.6 pg/ml (range: 0.5–19 833 pg/ml). In 113 patients (36.2%), NT‐proBNP levels were within normal range (0–125 pg/ml), 199 patients (63.8%) had elevated NT‐proBNP levels, and in 20 patients (6.4%), NT‐proBNP exceeded 2000 pg/ml. There was no significant correlation between NT‐proBNP levels and platelet counts (R = 0.103; *p* = .071), hemoglobin (R = 0.071; *p* = .213), or white blood count (WBC) (R = 0.110; *p* = .054). However, when analyzing patients with WBC ≤1.0 × 10^3^/μl and those >1.0 × 10^3^/μl, NT‐proBNP levels differed significantly (*p* < .001), although there was a relevant overlap in NT‐proBNP levels between these groups (Figure S2). Serum NT‐proBNP levels differed significantly between patients aged <60 years (median NT‐proBNP level: 146.7 pg/ml; range: 0.5–15 930 pg/ml) and patients ≥60 years (median NT‐proBNP level: 226 pg/ml; range 1–19 883 mg/dl; *p* = .003) and a weak correlation was observed between NT‐proBNP levels and age (R = 0.243; *p* = .001; Figure S3A). Moreover, we found a weak correlation between serum NT‐proBNP levels and serum lactate dehydrogenase (LDH) levels (R = 0.308; *p* < .001), NT‐proBNP levels and albumin (R = 0.168; *p* = .006), NT‐proBNP levels and CRP (R = 0.218; *p* = .000) and NT‐proBNP levels and serum creatinine concentrations (R = 0.244; *p* < .001) (Table S3 and Figure S3B,C).

The median serum NT‐proBNP levels was 199.5 pg/ml (range: 0.5–19 833 pg/ml) in patients with de novo AML and 151.6 pg/ml (range: 13.5–5115 pg/ml) in patients with secondary AML (*p* = .298). Serum NT‐proBNP levels were higher in patients with mutated *NPM1* (*NPM1*‐mut) as compared to those with *NPM1*‐wildtype (wt) (*p* < .001). Patients with *FLT3*‐internal tandem duplication (ITD) did not differ from those with *FLT3*‐wt (*p* = .348) regarding serum NT‐proBNP levels. Reports from echocardiography were available in 188 patients. Of these, 172 had a normal left ventricular function and 14 had a slightly or moderately reduced left ventricular function. The median NT‐proBNP level in patients with normal left ventricular function was 184.4 pg/ml (range 1–7393 pg/ml) and thus lower than in those with slightly to moderately reduced left ventricular function (988.6 pg/ml, range 161–19 833 pg/ml) (*p* < .001).

### 3.2 NT‐proBNP levels correlate with the CCI and ECOG performance status

In 293 patients, the medical history included information on comorbidities. The most frequently recorded comorbidities were infections (*n* = 120) followed by cardiac disorders (*n* = 54), history of a solid tumor (*n* = 42), and diabetes mellitus (*n* = 25). In 26 patients, a hematologic neoplasm, including myeloproliferative or myelodysplastic syndromes preceded the AML (Table S4). Patients presenting with cardiac and renal disorders had the highest median NT‐proBNP levels (2069.0 pg/ml; range 136–19 833 pg/ml) compared to NT‐proBNP levels in those with cardiac disorders (425.0 pg/ml; range: 17–5768 pg/ml), those with renal disorders (265.1 pg/ml; 12–955), patients with other comorbidities (163.0 pg/ml; 0.5–15 930), and those without recorded comorbidities (1431 pg/ml; 1–1799) (*p* < .001). According to the CCI, 59 patients (20.4%) had no comorbidity‐related risk, 79 (27.3%) were in the moderate, 95 (32.9%) in the intermediate, and 56 (19.4%) in the high‐risk group. The median NT‐proBNP levels differed significantly between patients with high risk (307.65 pg/ml), intermediate risk (199.50 pg/ml), moderate risk (156.00 pg/ml), or no risk according to the CCI (139.30) (*p* = .028) (Figure S4).

No significant difference in NT‐proBNP levels was observed between males (median: 188.0 pg/ml; range: 0.5–19 833) and females (median: 194.4 pg/ml; range: 1–15 930) (*p* = .328). Among all AML patients examined, five (1.6%) were regarded underweight (BMI < 18.5 kg/m^2^), 108 (34.6%) normal weight (BMI 18.5–24.9 kg/m^2^), 112 (35.9%) overweight (BMI 25.0–29.9 kg/m^2^), and 57 (18.3%) had obesity (BMI >30 kg/m^2^) at diagnosis without any significant difference when comparing NT‐proBNP levels in these subgroups (*p* = .767).^34^

The ECOG performance status was available in 310 patients. A majority of these patients (*n* = 214; 69.0%) were found to have a normal status (ECOG 0), 89 patients (28.7%) had ECOG 1, five patients (1.6%) ECOG2, and two patients (0.7%) ECOG 3. There was a significant difference in the median NT‐proBNP levels between the different ECOG groups (ECOG 0: 156.9 pg/ml, range: 1–5115 pg/ml; ECOG 1: 264.6 pg/ml, range: 0.5–15 930 pg/ml; ECOG 2: 6073, range: 1196–19 833 pg/ml; ECOG 3: 1331.8, range: 145.5–2518; *p* < .001).

### 3.3 NT‐proBNP is a prognostic marker for response to induction therapy and early death

Following induction therapy, 219 patients (70.2%) achieved a CR. In 63 patients, a blast cell persistence was found (no remission = NR; 20.2%), and 30 patients (9.6%) died within 60 days from the start of induction therapy (=ED). Median NT‐proBNP levels differed significantly among patients with CR, NR, and ED, amounting to 153.3 pg/ml (range 0.5–15 930 pg/ml), 225.2 pg/ml (5–5115 pg/ml), and 735.5 pg/ml (17–19 833 pg/ml), respectively (*p* < .001; Figure S5A). However, we also observed a substantial overlap between the median NT‐pro‐BNP values of AML patients achieving CR and non‐responders.

In multivariate analyses using a generalized linear model that included NT‐proBNP together with age, sex, the ELN‐2009 classification, WBC, albumin, LDH, CRP and CCI, the NT‐proBNP level at diagnosis remained an independent adverse prognostic factor predicting responses to induction chemotherapy (*p* = .009; Table 2). In this analysis, age as well as the ELN‐2009 classification were also found to be independent prognostic variables predicting response to induction therapy (*p* = .038 and *p* < .001, respectively).

**TABLE 2:** Prognostic factors for response to induction therapy

Marker	*p*‐value*
Age (years) (*n* = 312)	**.038**
Sex (male/female) (*n* = 312)	.055
NT‐proBNP (pg/ml) (*n* = 312)	**.009**
ELN‐2009 (*n* = 290)	**<.001**
WBC (10^3^/μl) (*n* = 312)	.446
CCI (*n* = 289)	.456
LDH (U/L) (*n* = 310)	.386
Albumin (g/L) (*n* = 270)	.154
CRP (mg/dl) (*n* = 312)	.728

*Note*: Bold values denotes the *p*‐values <0.05.

Abbreviations: CCI, Charlson comorbidity index; ELN‐2009, European leukemia net classification 2009; NT‐proBNP, NT pro BNP brain natriuretic peptide; WBC, white blood count.

* Multivariate analysis using a generalized linear model.

The management of AML differs between patients aged <60 years and those ≥60 years of age in clinical practice.^33^ In fact, the prevalence of frailty and medical comorbidities increase with age and chemotherapy‐related toxicity in the elderly is higher compared to younger patients.^33^ Likewise, severe neurotoxicity occurred in a significant number of patients aged ≥60 years receiving HiDAC.^4^ Moreover, NT‐proBNP levels differed significantly between patients aged <60 years. Therefore, we analyzed the difference in the median NT‐proBNP levels among patients with CR, NR, and ED separately in patients aged <60 years and those aged ≥60 years. In both age groups, the median NT‐proBNP levels differed significantly among patients with CR, NR, and ED (patients aged <60 years, *p* = .004; patients aged ≥60 years *p* = .001; Figure S5B,C). In patients with ED, infections (*n* = 11, 36.6%) were the most frequent causes of death, followed by bleedings (*n* = 6; 20.0%) and progression of disease (*n* = 4, 13.3%). The median NT‐proBNP levels were 672.5 pg/ml (range: 17–7393 pg/ml), 620 pg/ml (range: 52.1–4957), and 735.5 pg/ml (range 93.5–1085 pg/ml) in patients who that died from infections, bleedings, and progression of AML, respectively.

### 3.4 NT‐proBNP levels are of prognostic importance for survival

**FIGURE 1:**
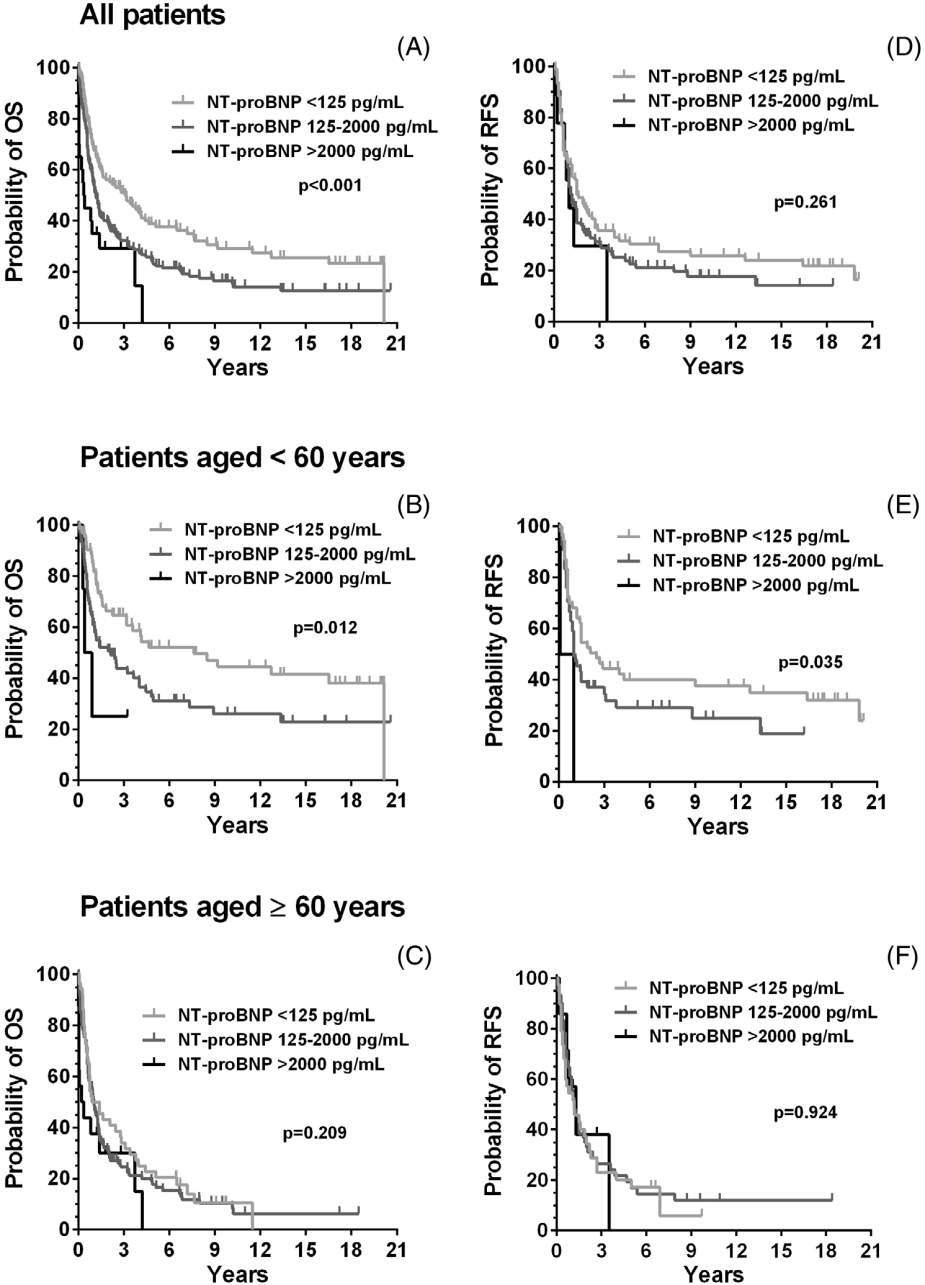
Survival in AML patients according to NT‐proBNP levels. Overall survival (OS) in the total cohort differed significantly among patients with normal NT‐proBNP (<125 pg/ml), those with moderately elevated (125–200 pg/ml), and those with highly elevated levels of NT‐proBNP (>2000 pg/ml) (*p* < .001 by log rank test) (A). Similar differences were observed in patients aged <60 years (*p* = .012) (B) but not in patients aged ≥60 years (*p* = .209) (C). The relapse‐free survival (RFS) did not differ significantly according to NT‐proBNP in all patients (*p* = .261) (D). In the cohort aged <60 years, a significant difference in RFS between patients with moderately (125–200 pg/ml) and highly (>2000) elevated NT‐proBNP (*p* = .035) (E) was seen, and this difference was not seen in those aged ≥60 years (*p* = .924) (F)

The median OS was 1.38 years with an interquartile range (IQR) of 0.96–1.84 in our cohort of AML patients. Significant differences were observed regarding OS between patients with normal (<125 pg/ml), moderately elevated (125–2000 pg/ml), and highly elevated NT‐proBNP (*p* < .001; Figure 1A). The median OS in these groups was 3.08 years (IQR 0.8–16.5), 1.14 years (IQR 0.5–4.8), and 0.34 years (IQR 0.04–3.7), respectively. In a multivariate analysis using Cox regression that included NT‐proBNP, age, the ELN‐2009 classification, creatinine levels, and the CCI, NT‐proBNP remained an independent prognostic variable for OS (*p* = .016; Table 3). In addition, the ELN‐2009 classification and age were independent prognostic factors in this analysis (*p* < .001 and *p* = .003, respectively).

**TABLE 3:** Prognostic factors of overall survival

Marker	Univariate analysis^a^, *p*‐value	Multivariate analysis^b^, *p*‐value
Age (yrs) (*n* = 312)	**<.001**	**.003**
Sex (male/female) (*n* = 312)	.475	
NT‐proBNP (pg/ml) (*n* = 312)	**<.001**	**.016**
ELN‐2009 (*n* = 290)	**<.001**	**<.001**
WBC (×10^3^/μl) (*n* = 312)	.70	
CCI (*n* = 289)	**<.001**	.642
Creatinine (mg/dl) (*n* = 274)	**.002**	.071
CRP (mg/dl) (*n* = 312)	.569	
Albumin (g/L) (*n* = 270)	.165	
LDH (U/L) (*n* = 310)	.062	

*Note*: Bold values denotes the *p*‐values <0.05.

Abbreviations: CCI, Charlson comorbidity index; ELN‐2009, European leukemia net classification 2009; NT‐proBNP, NT pro BNP brain natriuretic peptide; WBC, white blood count.

^a^ Univariate analysis using Cox regression.

^b^ Multivariate analysis using Cox regression.

In patients aged <60 years, the median OS was 3.2 years (0.8–20.2). Patients with normal, moderately elevated, and highly elevated NT‐proBNP levels had a median OS of 7.6 years (IQR 1.3–20.2), 2.1 years (IQR 0.6–13.3) and 0.3 years (IQR 0.3–09), respectively. In this group, NT‐proBNP levels were also found to be of prognostic significance (Figure 1B; *p* = .012). No significant differences in OS according to serum NT‐proBNP levels (normal, elevated, or very high) were observed in patients aged ≥60 years (Figure 1C; *p* = .20). After excluding patients with ED, NT‐proBNP remained to be prognostic for OS in all patients (*p* = .032) and in the group <60 years (*p* = .012). Similar results were obtained when analyzing OS after censoring HSCT (Figure S6A–C). Considering changing treatment regimens over time, we analyzed patients diagnosed between 1998 and 2007 and those diagnosed between 2008 and 2020 separately. In both groups, significant differences in OS according to serum NT‐proBNP levels (normal, elevated, or very high) were observed (see Table S5).

The median RFS of all AML patients analyzed was 1.3 years (IQR 0.6–2.7). No significant difference was observed between patients with normal, moderately elevated, and highly elevated NT‐proBNP levels (Figure 1D; *p* = .26). In patients aged <60 and ≥ 60 years, median RFS was 1.5 years (IQR 0.6–19.8) and 1.2 (IQR 0.5–2.7) years, respectively. In those aged <60 years, the RFS differed significantly between patients with normal NT‐proBNP (median RFS: 1.2 yrs; IQR 0.6–19.8), elevated NT‐proBNP (median RFS: 1.0 years, IQR 0.6–8.8) and highly elevated NT‐proBNP (median RFS: 0.1 years, IQR 0.1–1.0) (Figure 1E; *p* = .035). No differences in the survival of patients with normal, elevated and highly elevated NT‐proBNP were found in the group aged ≥60 years (Figure 1F). In the analysis of RFS after censoring patients with HSCT similar results were obtained (Figure S6).

## 4 DISCUSSION

Karyotype, genetic abnormalities, and patient‐related factors are of prognostic importance in patients with AML. Among patient‐related variables, a highly relevant factor is the presence of cardiac co‐morbidities. However, it is extremely difficult to estimate the risk of developing heart failure during chemotherapy in individual patients. The use of certain biomarkers, such as NT‐proBNP may represent a way to explore this risk. However, so far, little is known about the impact of NT‐proBNP levels regarding the outcome of AML patients. We found that NT‐proBNP is a significant and independent prognostic factor predicting early death and outcome after induction chemotherapy in AML patients aged <60 years and AML patients aged ≥60 years. Moreover, OS differed significantly between patients with normal, moderately elevated, and highly elevated NT‐proBNP levels. These differences in survival were primarily found in patients <60 years but not in patients aged ≥60 years.

NT‐proBNP is a biomarker broadly used for the diagnosis, evaluation, and monitoring of cardiomyopathies. In addition, NT‐proBNP is also known to serve as a useful marker for early detection of asymptomatic or imminent heart failure.^10^, ^11^, ^15^ Several studies have also shown that, apart from cardiac disorders, NT‐proBNP levels are also of prognostic significance in various other diseases. Examples are type 2 diabetes, severe septicemia, or amyloidosis.^16^, ^17^, ^18^, ^19^ There are also reports that point at the prognostic value of NT‐proBNP levels in hematologic disorders, including sickle cell disease and NHL.^20^, ^21^, ^22^, ^23^ In the current study, we demonstrate that NT‐proBNP is of prognostic significance in patients with AML. We found that the serum levels of NT‐proBNP differ significantly between AML patients achieving CR, NR, or ED. The cohort with ED had the highest NT‐proBNP levels at diagnosis. This elevation of NT‐proBNP may indicate an early asymptomatic phase of a cardiac disorder that might render these patients more sensitive for severe cardiac problems during induction chemotherapy. This is of particular importance since anthracyclines, used as components of induction therapy, are well‐known to be potentially cardiotoxic.^35^, ^36^ Elevated NT‐proBNP levels may, therefore, point at a specific vulnerability to toxic effects of such agents in patients with AML. This may explain the correlation between ED and elevated NT‐proBNP levels. An alternative hypothesis would be that elevated NT‐proBNP levels correlate with resistance of AML cells against cytotoxic drugs and thus with a poor outcome during induction therapy. This hypothesis would be supported by the observation that elevated NT‐proBNP levels are more frequently found in NR patients than in CR patients. In this regard, it is noteworthy that there is a substantial overlap in NT‐proBNP levels among patients with NR and those with CR. Moreover, there may be additional reasons for the correlations between NT‐proBNP levels and the outcome of induction therapy. For example, NT‐proBNP levels are also of prognostic value in patients with septicemia, independent of underlying cardiac disorders.^16^, ^37^, ^38^ In this regard, it is worth noting that AML patients with decreased cardiac function may have a higher probability to die during a severe infection, including bacterial septicemias which are commonly found in AML patients, especially during induction chemotherapy.^37^, ^38^, ^39^

It is also worth noting that NT‐proBNP levels are influenced not only by cardiac function but also by several other pathologic conditions. Especially, kidney disorders with reduced kidney function may lead to a substantial increase in NT‐proBNP levels.^40^, ^41^ Corresponding results were obtained in our study. In particular, the highest NT‐proBNP levels were found in patients suffering from both renal and cardiac disorders. Slightly lower, but still elevated, NT‐proBNP levels were found in AML patients with cardiac disorders without renal disease, followed by patients with renal dysfunction. The weak correlation between creatinine and NT‐proBNP levels suggests that cardiac dysfunction was the primary cause of elevated NT‐proBNP in our patients and a moderately decreased renal function, observed in most of these cases, just amplified the NT‐proBNP‐elevating effect of the cardiopathy.

In previous studies, elevated NT‐proBNP has been described as a predictor of poor survival in NHL.^36^, ^38^ In these publications, the prognostic significance of NT‐proBNP was independent of other variables, including comorbidities, physical performance status, and NHL specific disease‐related risk factors. Similarly, in our patients with AML, NT‐proBNP was not only of prognostic value for the outcome of induction therapy but also for OS. As NT‐proBNP is a patient‐specific marker, it may serve as a predictor of tolerability of chemotherapy but also as a marker for survival.

In this study, high serum NT‐proBNP was an unfavorable prognostic marker for survival, independent of age, sex, WBC, the ELN‐2009 classification, and CCI. Moreover, NT‐proBNP remained of prognostic significance for OS after excluding patients with ED from the survival analysis. Interestingly, the prognostic significance of NT‐proBNP concerning OS and RFS was primarily observed in the cohort aged <60 years. When only examining elderly patients, NT‐proBNP was not of prognostic significance for OS and RFS. This is in contrast with the above reported results obtained with induction therapy where NT‐proBNP was a prognostic factor for the outcome in patients aged <60 years and patients aged ≥60 years. However, similar differences of the prognostic value of NT‐proBNP levels among patients aged 60 years and ≥ 60 years regarding survival have also been reported by Haas et al.^41^ In patients undergoing HSCT, the prognostic significance of NT‐proBNP in patients undergoing HSCT was restricted to the cohort aged <60 years.^42^ In patients ≥60 years, no significant difference regarding 100‐day mortality was observed.^42^

LDH and albumin are generally accepted prognostic variables for survival in cancer patients and might mask the effect of NT‐proBNP with regard to long‐term survival but not with regard to the short‐term outcome of induction therapy.^43^, ^44^, ^45^, ^46^ Both markers, together with platelet counts improved the prognostic value of comorbidity scores significantly, as shown by Sorror et al.^47^ In our analyses, the correlations between LDH and NT‐proBNP levels and between albumin levels and NT‐proBNP levels were significant, which highlights the importance of these markers. Whether changes in albumin and LDH levels directly relate to pathologies upregulating NT‐proBNP levels remains unknown. Some of the relevant co‐morbidities, including cardiac pathologies, may indeed induce changes in LDH and albumin levels as well as an elevated NT‐proBNP.

The discrepancy in the prognostic value of NT‐proBNP between patients aged <60 years and ≥ 60 years might also be explained by the fact, that elderly patients in general have a higher risk to be more heavily health‐compromised and to present with various confounding parameters.^26^ NT‐proBNP, as a rather unspecific marker loses its significance in this context. Thus, it is tempting to speculate, that an age‐related decrease in various organ functions is responsible for the increases of NT‐proBNP in these patients. In line with this assumption, NT‐proBNP levels correlated significantly with the number and severity of comorbidities as assessed by the CCI. In particular, the median NT‐proBNP levels were low in patients without essential comorbidities but higher in those with moderate, intermediate, and high‐risk comorbidities. In line with this observation, patients with a decreased performance status, typically seen in the elderly, had significantly higher NT‐proBNP levels compared to those with a normal performance status (ECOG 0). In contrast, in younger patients without comorbidities, NT‐proBNP may point to the presence of an occult cardiac dysfunction.

When considering NT‐proBNP levels as a new prognostic biomarker in patients with AML, several limitation have to be considered. First, AML patients collected during the past two decades have been included in this retrospective study. During this long period, treatment strategies and supportive care have in part changed. To address this issue, we analyzed patients treated until July 2008 and those treated thereafter separately in a subanalysis. In these analyses, the NT‐proBNP level remained a prognostic variable in both cohorts. Therefore, we considered NT‐proBNP levels to be of prognostic value in patients with AML independent of the time (year) when therapy had been applied. Secondly, in many patients, NT‐proBNP levels were assessed after the serum had been stored for up to 22 years at −20°C. Previous data have shown that that NT‐proBNP levels in stored samples at −20°C remain stable for at least 2 years.^48^ We compared the median NT‐proBNP levels of our AML patients in whom serum was analyzed at the day of sampling with those in whom NT‐proBNP levels were determined in samples stored for 4–5 years, >5–10 years, >10–15 years, or >15–22 years at −20°C. In this analysis, NT‐proBNP levels were within the same ranges without a significant decrease in median NT‐proBNP levels over time. Finally, since 2016, NT‐proBNP was measured at diagnosis in our AML patients because it had been introduced as routine test at our center in January 2016. Although NT‐pBNP measurements were soon regarded as important and useful in the evaluation of the cardiac status in our patients, we still based our treatment decision (regarding cardiac function) on echocardiography findings (not on NT‐proBNP levels) following internal rules and international guidelines.^31^

Together, NT‐proBNP is an independent prognostic factor and new biomarker in the context of AML indicating the risk of induction failure and ED regardless of age. NT‐proBNP is also of prognostic importance regarding OS, primarily in patients aged <60 years. Thus, NT‐proBNP at diagnosis provides independent and powerful information to predict induction failure, ED, and survival in AML patients undergoing intensive therapy.

## AUTHOR CONTRIBUTIONS

IG, PV, and WRS contributed to the study design, data collection and analysis, and drafting of the manuscript. MK performed statistical analyses. GG, GS, RM, GH, GMH, IS, and RT contributed to material collection and analysis. KVG, SH, PK, UJ, and AH contributed to data collection. All authors critically reviewed the manuscript and approved the final version of the document.

## CONFLICT OF INTEREST

IG, GG, RM, KVG, SH, GS, PK, UJ, AH, IS, RT, MK, GH, GMH, PV, and WRS do not have any competing financial interests in relation to the work described.

## PATIENT CONSENT

All patients provided written informed consent.

## Supporting information


**Appendix S1.** Supporting Information

## Data Availability

The data that support the findings of this study are available on request from the corresponding author.
