# Supplementary material for: N‐terminal pro‐brain natriuretic peptide is a prognostic marker for response to intensive chemotherapy, early death, and overall survival in acute myeloid leukemia
Source: Am J Hematol. 2023 Jan 1;98(2):290–9. doi: 10.1002/ajh.26805 (PMC10107495; doi:10.1002/ajh.26805)
Supplement: Supplementary file 1 — Appendix S1. Supporting Information [file AJH-98-290-s001.docx]

**Supplementary Material:**

**N-terminal Pro-Brain Natriuretic Peptide is a Prognostic Marker for Response to Intensive Chemotherapy, Early Death, and Overall Survival in Acute Myeloid Leukemia**

Irene Graf, M.D.^1^, Georg Greiner, M.D.^2,3,4^, Rodrig Marculescu, M.D.^2^, Karoline V. Gleixner, M.D.^1^, Susanne Herndlhofer^1^, Gabriele Stefanzl^1,3^, Paul Knoebl, M.D.^1^, Ulrich Jäger, M.D.^1,5^, Alexander Hauswirth, M.D.^1^, Ilse Schwarzinger, M.D.^2^, Renate Thalhammer, M.D.^2^, Michael Kundi, M.D.^6^, Gregor Hoermann, M.D.^3,7^, Gerlinde Mitterbauer-Hohendanner, M.D.^2^, Peter Valent, M.D.^1,3,5^, Wolfgang R. Sperr, M.D.^1,3,5^

^1^ Department of Internal Medicine I, Division of Hematology and Hemostaseology, Medical University of Vienna, Vienna, Austria; ^2^Department of Laboratory Medicine, Medical University of Vienna, Vienna, Austria; ^3^Ludwig Boltzmann Institute for Hematology and Oncology, Medical University of Vienna, Vienna, Austria; ^4^Ihr Labor, Medical Diagnostic Laboratories, Vienna, Austria; ^5^Comprehensive Cancer Center Vienna, Medical University of Vienna, Vienna, Austria ^6^Institute of Environmental Health, Medical University of Vienna, Vienna, Austria; ^7^MLL Munich Leukemia Laboratory, Munich, Germany

| **Supplementary Table 1**  **Chemotherapy protocols applied in patients with AML (n= 312)** | | |
| --- | --- | --- |
| Induction therapy | | |
| All patients | 1998 to October 2016 | After October 2016 |
|  | DAV 3+5+7 (daunorubicin, 45 mg/m² iv, day 1-3; etoposide, 100 mg/m² iv, day 1-5; ARA-C 2 x 100 mg/m² iv, day1-7) | DA 3+7 (daunorubicin 60 mg/m² iv, day 1-3, ARA-C 100 mg/m² ci, day 1-7) |
| 2nd Induction* | | |
| Patients aged <60 years | MiDAC (ARA-C, 2 x 1 g/m² iv, day 1-4, mitoxantron 12 mg/m², day 3-5) | |
| Patients aged ≥60 years | 1998 to July 2008 | After July 2008 |
|  | DAV 2+5+5 (daunorubicin, 45 mg/m² iv, day 1-2; etoposide, 100 mg/m² iv, day 1-5; ARA-C 2 x 100 mg/m² iv, day 1-5) | MiDAC (ARA-C, 2 x 1 g/m² iv, 3 day 1,3,5; mitoxantrone, 12 mg/m² iv, 2 day 3 & 5) |
| 1^st^ Consolidation | | |
| Patients aged <60 years | 1998 to February 2016 | After February 2016 |
|  | HiDAC (2 x 3 g/m^2^ iv, day ARA-C iv day 1,3,5 or day 1,2,3) | FLAG (fludarabine, 30 mg/m² iv, day 1-5; ARA-C. 2 g/m² iv, days 1-5; G-CSF, sc day 6 until recovery) |
| Patients aged ≥60 years | 1998 to July 2008 | After July 2008 |
|  | IDAC (2 x 1 g/m^2^ iv, day ARA-C iv, day 1,3,5 or day 1,2,3) | FLAG (fludarabine, 30 mg/m² iv, day 1-5; ARA-C. 2 g/m² iv, day 1-5; G-CSF, sc day 6 until recovery) |
| 2^nd^ to 4^th^ Consolidation | | |
| Patients aged <60 years | HiDAC (2 x 3 g/m^2^ iv, day ARA-C iv day 1,3,5 or day 1,2,3) | |
| Patients aged ≥60 years | IDAC (2 x 1 g/m^2^ iv, day ARA-C iv, day 1,3,5 or day 1,2,3) | |
| Abbreviations: ci, continuous infusion; HiDAC, high dose ARA-C; IDAC, intermediate dose ARA-C; iv, intravenously; sc, subcutaneously; G-CSF, granulocyte colony-stimulating factor. * In case of blast cell persistence. | | |

| **Supplementary Table 2**  **Comorbidity-related scoring at diagnosis in patients with** | | |
| --- | --- | --- |
|  |  | n (%) |
| ECOG (n=310) | 0 – normal activity | 214 (69.1%) |
|  | 1 – mild symptoms | 89 (28.7%) |
|  | 2 – symptoms, but in bed <50% | 5 (1.6%) |
|  | 3 - in bed >50%, but not bedridden | 2 (0.6%) |
|  | 4 - Unable to get out of bed | 0 (0.0%) |
|  | 5 - dead | 0 (0.0%) |
| CCI (n=289) | No comorbidities* | 59 (20.4%) |
|  | Low risk | 79 (27.3%) |
|  | Moderate risk | 95 (32.9%) |
|  | High risk | 56 (19.4%) |
| CCR, Charlson comorbidity index; ECOG, Eastern Cooperative Oncology Group; n, number of patients; *, according to CCI | | |

**Supplementary Table 3**

| **NT-proBNP levels according to the patients’ characteristics** | | |
| --- | --- | --- |
| NT-proBNP | Median (IQR)  NT-proBNP level (pg/mL) | p-value* |
| WBC (×10^3^/μL) (n = 312) |  |  |
| - ≤ 10 ×10^3^/μL | 122.4 (281.8) | < 0.001 |
| > 10 ×10^3^/μL | 340.9 (643) |  |
| Platelets (×10^3^/μL) (n = 312) |  |  |
| ≤ 150 ×10^3^/μL | 198 (561) | 0.387 |
| >150 ×10^3^/μL | 170.6 (289) |  |
| Hemoglobin (g/dL) (n = 312) |  |  |
| - ≤ 8mg/dl | 196.4 (550) | 0.758 |
| > 8mg/dl | 181.4 (507) |  |
| Age (yrs) (n = 312) |  |  |
| - ≤ 60a | 146.7 (374) | 0.003 |
| > 60a | 226 (669) |  |
| Creatinine (mg/dl) (n = 274) |  |  |
| - ≤ 1,2mg/dl | 172.6 (419) | 0.004 |
| > 1,2 mg/dl | 572 (1676) |  |
| CRP (mg/dL) (n = 312) |  |  |
| - ≤ 0,5 mg/dl | 118.7 (162) | < 0.001 |
| - > 0,5 mg/dl | 262 (601) |  |
| LDH (U/L) (n = 310) |  |  |
| < 240 U/L | 91.3 (313) | 0.002 |
| > 240 U/L | 226 (595) |  |
| Albumin (g/L) (n = 270) |  |  |
| - ≤ 35 mg/dl | 489.4 (975) | 0.001 |
| - > 35 mg/dl | 171.3 (381) |  |
| Abbreviations: NT-proBNP, NT pro BNP brain natriuretic peptide; CRP, C-reactive protein; WBC, white blood cell count; LDH, lactate dehydrogenase; IQR; interquartile range; AML, acute myeloid leukemia; *p-value assessed by univariate analysis (Mann Whitney Test) | | |

| Supplementary Table 4  Comorbidities at diagnosis in patients with AML | | |
| --- | --- | --- |
|  |  | n (%) |
| Comorbidity* | Infection | 120 (41.5%) |
| n=289 | Cardiac disease | 54 (18.7%) |
|  | Solid tumor | 42 (14.5%) |
|  | Prior hematologic malignancy | 26 (9.0%) |
|  | Diabetes mellitus | 25 (8.7%) |
|  | Hepatic disease | 22 (7.6%) |
|  | Obesity | 19 (6.7%) |
|  | Pulmonary disease | 18 (6.2%) |
|  | Psychiatric disease | 17 (5.8%) |
|  | Peptic ulcer | 15 (5.1%) |
|  | Peripheral vascular disease | 12 (4.2%) |
|  | Renal disease | 12 (4.2%) |
|  | CVD or TIA | 11 (3.8%) |
|  | Rheumatologic disease | 8 (2.8%) |
|  | Lymphoma | 3 (1.0%) |
|  | Inflammatory bowel disease | 2 (0.7%) |
|  | Hemiplegia | 1 (0.3%) |

Abbreviations: AML, acute myeloid leukemia; CVD, cerebral vascular disease; n, number of patients; TIA, transitory ischemic attack

**Supplementary Table 5**

**Overall survival in patients with AML 1998-2007 and 2008-2020**

| Variable | Median overall survival | |
| --- | --- | --- |
|  | 1998-2007, n=178 (p=0.002*) | 2008-2020, n=134 (p=0.043*) |
| OS in all patients – overall OS | 1.16 | 2.07 |
| OS in patients with normal NT-proBNP | 2.76 | 3.58 |
| OS in patients with elevated NT-proBNP | 1.08 | 1.37 |
| OS in patients with highly elevated NT-proBNP levels | 0.39 | 0.34 |
| Abbreviations: AML, acute myeloid leukemia; n, number of patients; NT-proBNP, N-terminal Pro-Brain Natriuretic Peptide; OS, overall survival. *We found significant differences in OS among patients with normal, elevated or highly elevated NT-proBNP levels as determined by the log rank test. | | |

**Supplementary Figure 1:**

**Median NT-proBNP levels according to the time of measurement**

Serum samples were obtained from 312 patients with AML and were either measured immediately or after having been stored in a local biobank for 4-5 years, >5-10 years, >10-15 years, or >15-22 years at -20°C. After freeze-thawing, the NT-proBNP levels were quantified as described in the section ´Patients and Methods´. The NT-proBNP levels measured in these 5 sub-groups were within the same ranges and, most importantly, no significant decrease in median NT-proBNP levels could be observed when comparing samples stored over different time periods (p=0.169 by ANOVA). The lines represent the median values, the whiskers show the interquartile ranges.

**Supplementary Figure 2:**

**Serum NT-proBNP levels in AML patients with a normal white blood count (WBC) (left panel) or elevated WBC count (right panel) at diagnosis**

Serum NT-proBNP levels were determined in 312 patients with AML as described in the section ´Patients and Methods´. There was a significant difference in the median NT-proBNP level obtained in AML patients with a WBC ≤ 10 G/L (median serum NT-proBNP level: 122.4 pg/ml) and those AML patients in whom a WBC > 10 G/L was found (median NT-proBNP level: 340.9.pg/ml; p<0.001 as assessed by Mann-Whitney test). However, we also observed a relevant overlap between the NT-proBNP levels among these groups was observed. The lines represent the median values and the whiskers the interquartile ranges.

**Supplementary Figure 3:**

**Correlation between serum NT-proBNP levels and other variables in patients with AML**

Serum NT-proBNP levels were determined in 312 (age), 310 (lactate dehydrogenase=LDH), or 270 (creatinine) patients with AML as described in the the section ´Patients and Methods´, respectively. As assessed by a linear regression model, we found weak correlations between NT-proBNP levels and age (A), NT-proBNP levels and LDH (B), and NT-proBNP levels and serum creatinine concentrations (C). Correlation coefficients (R) as determined by the linear regression model: R=0.243 with p=0.001 (A); R=0.308 with p<0.001 (B), and R=0.244 with p<0.001 (C).

**Supplementary figure 4:**

**Serum NT-proBNP levels in different risk groups defined by the Charlson comorbidity index (CCI) in the patients with AML**

Serum NT-proBNP levels were determined in 289 patients with AML as described in the the section ´Patients and Methods´. NT-proBNP concentrations differed significantly among patients in different groups defined by the CCI. The highest level was found in CCI group with ≥5 risk factors, followed by patients with 3-4, 1-2, and 0 risk factors (p=0.003 as assessed by Kruskal Wallis test). The lines represent the median NT-proBNP values, and the whiskers show the interquartile ranges. Asterisks: significant differences in NT-proBNP levels among two groups with p<0.05 as assessed by the Mann Whitney test.

**Supplementary figure 5:**

**NT-proBNP levels in AML patients according to the outcomes after induction poly-chemotherapy**

NT-proBNP levels were measured in all patients (2A), in those aged <60 years (2B), and in those aged ≥60 years (2C). Serum NT-proBNP levels were determined in 312 patients with AML as described in the the section ´Patients and Methods´. The median NT-proBNP levels differed significantly among patients with complete remission (CR), no remission (NR), and early death (ED) in all three groups as assessed by Kruskal Wallis test). The highest NT-proBNP levels were found in patients with ED followed by patients with NR and those with CR. The lines represent the median, the whiskers the interquartile range. Asterisk denotes significant differences between two groups as assessed by the Mann Whitney test. The lines represent the median, the whiskers the interquartile ranges.

**Supplementary figure 6:**

**Survival in AML patients according to NT-proBNP levels after censoring for allogeneic hematopoietic stem cell transplantation**

Serum NT-proBNP levels were determined in 312 patients with AML as described in the the section ´Patients and Methods´. Overall survival (OS) in the total cohort differed significantly among patients with normal NT-proBNP levels (<125 pg/mL), those with moderately elevated NT-proBNP levels (125-200 pg/mL), and those with highly elevated NT-proBNP levels (>2000 pg/mL) (p<0.001 by log rank test) (A). Similar differences were observed in the subgroup of patients aged <60 years (p=0.006) (B) but not in patients aged ≥60 years (p=0.231) (C). Relapse-free survival (RFS) did not differ significantly when comparing patients with higher or lower NT-proBNP levels in the total cohort of (all) patients (p=0.369) (D). In the cohort aged <60 years, a significant difference between patients with moderately (125-200 pg/mL) and highly (>2000) elevated NT-proBNP was found (p=0.012) (E) but this difference was not found in patients aged ≥60 years (p=0.846) (F).
